# Disorder of the immune and inflammatory response involving galectin-1, -3 and -7 in women with invasive breast cancer – potential importance in diagnosis and monitoring the course of the disease

**DOI:** 10.3389/fonc.2026.1773190

**Published:** 2026-03-12

**Authors:** Patrycja Królewska-Daszczyńska, Jacek Kabut, Celina Kruszniewska-Rajs, Joanna Magdalena Gola, Monika Paul-Samojedny, Aleksandra Mielczarek-Palacz

**Affiliations:** 1 Department of Immunology and Serology, Faculty of Pharmaceutical Sciences in Sosnowiec, Medical University of Silesia, Katowice, Poland; 2 Department of Oncology and Radiotherapy, Medical University of Silesia, Katowice, Poland; 3 Department of Molecular Biology, Faculty of Pharmaceutical Sciences in Sosnowiec, Medical University of Silesia, Katowice, Poland; 4 Department of Medical Genetics, Faculty of Pharmaceutical Sciences in Sosnowiec, Medical University of Silesia, Katowice, Poland

**Keywords:** breast cancer, galectin-1, galectin-3, galectin-7, immune disorder, inflammatory response

## Abstract

**Purpose:**

As implications of galectins in cancer biology have been proved, it is essential to understand their role in immune and inflammatory response as well as investigate their potential clinical application. Therefore, the aim of this study was a comprehensive analysis of serum concentration and mRNA expression of galectins in patients with invasive breast cancer (BC).

**Methods:**

Serum concentration of galectin-1, -2, -3, -4, -7, -8 and -9 in 60 women with invasive BC and 20 women with benign lesions were determined using the enzyme-linked immunosorbent assays (ELISA). The mRNA expression levels were assessed using real-time RT-qPCR reactions.

**Results:**

The results showed significantly increased concentrations of galectin-3 and -7 in BC patients compared to control group. Moreover, significantly elevated concentrations of galectin-1, -3, -4, and -7 were observed in luminal BC subtypes. Then, the analysis at the transcript level performed for galectin-1, -3 and -7 revealed no significant differences in the expression of *LGALS1* and *LGALS7* mRNA levels between BC and control group. The expression of *LGALS3* mRNA were not shown neither for BC patients, nor control group. Additionally, the combinatorial analysis of galectins with classic markers CA15–3 and CRP showed that combinations with galectin-1, -3 and -7 may improve the diagnostic performance of single classical markers in discriminating BC from benign lesions.

**Conclusions:**

The results of the study may confirm the importance of galectins in the immune response and the development of inflammatory reactions in BC. The combination of galectin-1, -3 and -7 measurements with conventional markers has the potential to facilitate the differential diagnosis of patients with breast cancer and benign lesions. Furthermore, it may hold significance in the monitoring of the inflammatory process during the course of the disease. The demonstrated presence of galectins derived from cancer cells in the blood of women with breast cancer provides a basis for further research into their use as potential biomarkers, useful both in diagnosis and monitoring of the course of the disease and also creates opportunities for application in modern targeted therapy.

## 1 Introduction

Despite advances in early detection and treatment breast cancer (BC) remains the most common malignancy and the leading cause of cancer-related deaths among women worldwide (1). The global differences in BC incidence and survival rates are associated with diverse possibilities of screening that enable early diagnosis and access to appropriate treatment, but also with different reproductive and lifestyle risk factors (1, 2). The diagnosis of BC is based on imaging techniques, such as mammography, ultrasound or magnetic resonance imaging (MRI), and a tumour biopsy for histopathological and molecular analysis (3, 4).

Molecular subtyping based on estrogen receptor (ER), progesterone receptor (PR), human epidermal growth factor receptor-2 (HER-2) and Ki67 expression identifies luminal A, luminal B, HER2-enriched and triple-negative BC (TNBC) which differ in prognosis and therapeutic response (3, 5). Luminal A cancers, which represents the most common subtype, have more favourable prognosis and better response to endocrine therapy than luminal B tumours that can be divided according to HER2 status. Non-luminal HER2-enriched cancers as well as TNBC are associated with poor prognosis (4–8). Currently, numerous studies focus on potential minimally invasive biomarkers, especially blood-based biomarkers, that could be useful in BC diagnosis, monitoring of disease progression and treatment response.

Galectins are β-galactoside-binding proteins that modulate cell adhesion, immune regulation and apoptosis (9–12). Galectins are expressed in various tissues but also immune cells, epithelial and endothelial cells as well as neurons. They are synthesised in the cytoplasm and then distributed to various subcellular locations as well as they can be secreted extracellularly (13). Increasing evidence implicates specific galectins (e.g. galectin-1, -3, -9) in tumour progression, metastasis and immune evasion (14). The most extensively studied galectins, which have been linked to cancer biology are galectin-1, -3 and -9 (15, 16).

While individual galectins have been studied in several malignancies, a comprehensive analysis of circulating and transcriptional profiles of galectins-1, -2, -3, -4, -7, -8 and -9 in BC has not yet been performed. This study aims to fill this gap by evaluating both serum concentration and mRNA expression levels of these galectins across molecular BC subtypes. Such a comprehensive analysis will not only allow for a deeper understanding of the role of studied galectins in the regulation of immune and inflammatory response but also will allow indicate their use in improvement of laboratory diagnostic tools used for this cancer. Therefore, the aim of this study was the assessment of mRNA expression in blood and serum concentrations of galectins-1, -2, -3, -4, -7, -8 and -9 in patients with invasive BC including molecular subtypes and cancer grade as well as determining if there is a relationship between the studied parameters with classic markers used in laboratory diagnostics.

## 2 Materials and methods

### 2.1 Study design and participants

The patients included in the study were diagnosed at the Oncology Outpatient Clinic of the Regional Specialist Hospital No. 3 in Rybnik due to the detection of a solid breast lump using imaging techniques: breast ultrasound and mammography. Then, the patients were referred for core needle biopsy of the breast nodule and laboratory tests. Imaging, histopathological examination and laboratory tests were performed at the Diagnostics Centre of Regional Specialist Hospital No. 3 in Rybnik. Women who had previously undergone oncological treatment or had been diagnosed with autoimmune disease were excluded from the study.

#### 2.1.1 Study group

The study group included 60 female patients aged 35 – 87 (65.58 ± 11.76) with invasive BC confirmed in histopathological examination. The results of the histopathological examination included additional information on tumour histological type and grade (G1, G2, G3) as well as receptor status (expression of ER, PR and HER2) and the expression of the Ki67 proliferation index. Moreover, based on a clinical data, the disease stage was assessed according to the TNM classification. On the basis of molecular features included in the histopathological protocol, the patients were classified into one of the following subtypes of BC: Luminal A (n = 16), luminal B HER2- (n = 19), luminal B HER2+ (n = 9), non-luminal HER2+ (n = 5) and TNBC (n = 11). Patients characteristics are performed in **Table 1**.

**Table 1:** Clinicopathologic characteristics of BC patients.

Characteristic	Number of patients
T stage	
T1	23
T2	28
T3	4
T4	5
N stage	
N0	24
N1	25
N2	7
N3	3
unknown	1
M	
M0	46
M1	14
G (grade)	
G1	11
G2	28
G3	21

#### 2.1.2 Control group

The control group included 20 patients aged 27 – 61 (43 ± 9.33) with a benign breast nodule confirmed in histopathological examination. Based on the results of the histopathological examination, patients in the control group were diagnosed with benign breast lesions: eight patients with fibroadenoma and twelve patients with fibrosclerosis.

The statistical analysis showed that mean age of BC patients was significantly higher compared to patients with benign breast disease.

The study was conducted according to the guidelines of the Declaration of Helsinki and approved by the Ethics Committee of Medical University of Silesia in Katowice, Poland (protocol code PCN/CBN/0022/KB1/75/21). All participants provided their written informed consent prior to inclusion in the study.

### 2.2 Sample collection and processing

The venous blood was collected from all participants in a tube without any anticoagulant and in a tube containing K_2_EDTA. After 30 min the collected tube with no anticoagulant was centrifuge (1500 x g for 15 min) and the obtained serum was then portioned and frozen at −80 °C. The tubes with the whole blood was stored at the same temperature. All blood samples were collected before initiating therapeutic procedures.

### 2.3 Determination of galectin concentrations

#### 2.3.1 ELISA tests

Serum concentrations of galectin-1, -2, -3, -4, -7, -8 and -9 were determined *via* sandwich ELISA immunoenzymatic assays using CLOUD-CLONE ELISA kits for *in vitro* quantitative measurements of Galectin-1 (GAL1), Galectin-2 (GAL2), Galectin-3 (GAL3), Galectin-4 (GAL4), Galectin-7 (GAL7), Galectin-8 (GAL8) and Galectin-9 (GAL9) (Cloud-Clone Corp, USA). The sensitivity of these tests were accordingly typically less than 0.123 ng/ml, 0.62 ng/ml, 0.054 ng/ml, 0.124 ng/ml, 12.3 pg/ml, 0.063 ng/ml, 33 pg/ml. The characteristics of ELISA kits used in this study are shown in **Table 2** based on the protocols provided by manufacturer.

**Table 2:** The specifications of the tests used in this study.

Parameter	Unit	Sensitivity	Detection range
Galectin-1	ng/mL	0.123	0.312-20
Galectin-2	ng/mL	0.62	1.56-100
Galectin-3	ng/mL	0.054	0.156-10
Galectin-4	ng/mL	0.124	0.312-20
Galectin-7	pg/mL	12.3	31.2-2000
Galectin-8	ng/mL	0.063	0.156-10
Galectin-9	pg/mL	33	78-5000

### 2.4 mRNA expression analysis

#### 2.4.1 RNA extraction

Total RNA was isolated from the whole blood using Fenozol reagent (A&A Biotechnology, Poland) with the addition of precipitation enhancer to the aqueous phase (A&A Biotechnology, Poland) according to the manufacturer’s protocol. Concentration of RNA was quantified using spectrophotometer MaestroNano MN-913 (MaestroGen Inc, USA).

#### 2.4.2 Quantitative RT-qPCR

The mRNA levels of selected genes were assessed using real-time RT-qPCR reactions. First, the cDNA synthesis was performed with use of smART Combo First Strand cDNA Synthesis Kit for RT-qPCR (EURx, Poland) according to the manufacturer’s protocol. Fast SG qPCR Master Mix (2x) (EURx, Poland) was used for the qPCR reactions. The primers used in reaction (KiCqStart® Primers, Merck, Germany) sequences are shown in **Table 3**. The real-time qPCR reaction was performed using a LightCycler^®^ 480 System (Roche, Switzerland). All samples were tested in two technical replications. The specificity of the PCR reaction was evaluated with the melting temperature curve analysis and using electrophoresis of the amplification products in a 2% agarose gel. The mRNA expression level were then determined by the 2−ΔCt relative quantification method. Human glyceraldehyde 3-phosphate dehydrogenase (GAPDH) was used as a reference gene (internal control).

**Table 3:** The primers used in reaction sequences.

Gene name	Forward	Reverse	Product size	KiCqStart Primer Pair ID
ACTB	GATCAAGATCATTGCTCCTC	TTGTCAAGAAAGGGTGTAAC	191	H_ACTB_2
GAPDH	CTTTTGCGTCGCCAG	TTGATGGCAACAATATCCAC	139	H_GAPDH_2
LGALS1	AGTTAAAAGGGTGGGAGC	CACAAGCCATGATTGAGTC	107	H_LGALS1_1
LGALS3	TGATTGTGCCTTATAACCTG	ATGACTCTCCTGTTGTTCTC	172	H_LGALS3_2
LGALS7	GGTTCCATGTAAACCTGC	CTCCTTGCTGTTGAAGAC	104	H_LGALS7B_1

### 2.5 Statistical analysis

The normality of distributions of the studied parameters was assessed using a Shapiro-Wilk test. To describe the obtained data the median of lower and upper quartiles was used. For a comparison of the two groups we used a non-parametric Mann-Whitney U test, while for a larger number of groups we used a Kruskal-Wallis ANOVA. The correlations were determined using a Spearman’s rank correlation test.

The age variable presents a normal distribution and is described as mean ± standard deviation (m ± SD). To compare the ages of breast cancer and benign groups we used a Student’s *t*-test.

The analysis were performed using Statistica software version 13 (StatSoft, TIBCO Software Inc.).

## 3 Results

### 3.1 Serum concentrations of galectins

The serum concentrations of galectin-1, -2, -3, -4, -7, -8 and -9 were determined in the breast cancer patients and the control group. The results were obtained for galectin-1, -3, -4, -7 and -9. For results below the detection range of a particular test, the exact values determined by the analyser were assumed, while for results below the test sensitivity the value 0 were assigned for statistical analysis. For four patients galectin-7 concentrations were above the detection range of the test, therefore for statistical analysis such results were assigned a concentration of the highest standard, which was 2000 pg/ml. The concentrations of galectin-2 were obtained only for four BC patients and non-patients from control group. For all women the concentrations of galectin-8 were below the test sensitivity. For these reasons, galectin-2 and -8 were excluded from further analysis.

The analysis of the results showed significantly higher serum concentrations of galectin-3 and -7 in patients with invasive BC compared to the control group (*p* < 0.05). No statistically significant differences in concentrations of other galectins were observed. **Table 4** presents the obtained results as a median with a lower and upper interquartile range (Q_1_-Q_2_).

**Table 4:** Serum concentration of galectins in patients with invasive BC and control group.

Parameter	Invasive BC (n=60)	Control group (n=20)	P-value
Galectin-1 [ng/mL]	0.45 (0.06-0.84)	0.06 (0.06-0.50)	*p* = 0.06
Galectin-3 [ng/mL]	1.75 (1.23-2.52)	1.33 (0.59-2.04)	*p* = 0.029*
Galectin-4 [ng/mL]	0.61 (0.00-2.43)	0.70 (0.00-1.74)	*p* = 1.0
Galectin-7 [pg/mL]	582.16 (504.57-767.13)	469.60 (127.40-628.55)	*p* = 0.004*
Galectin-9 [pg/mL]	36.19 (35.1-39.90)	37.55 (36.03-42.35)	*p* = 0.182

Data are presented as median (Q_1_-Q_3_); *p < 0.05 statistically significant.

#### 3.1.1 Serum concentration of galectin-1

**Figure 1:**
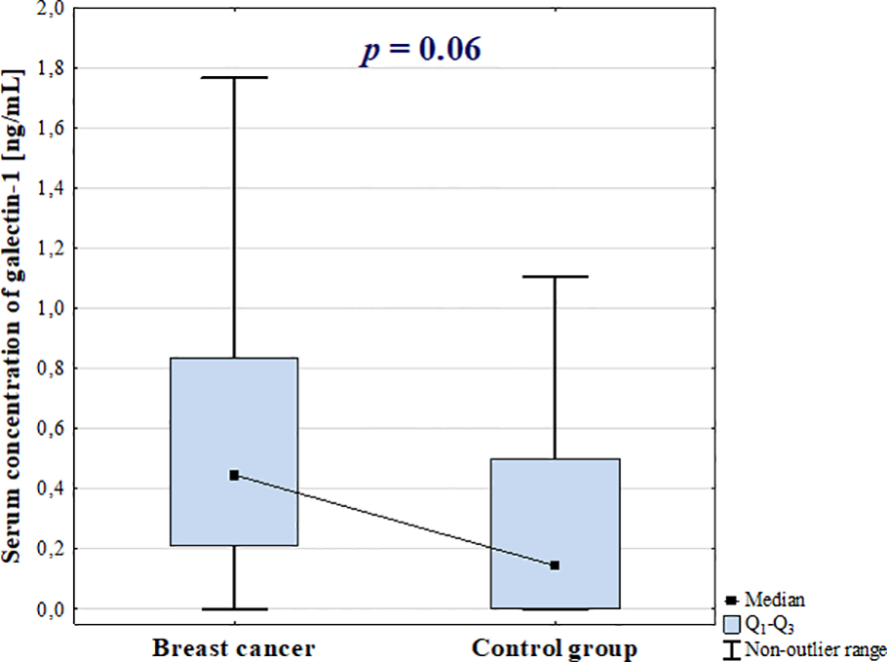
Serum concentrations of galectin-1 in breast cancer patients and control group.

Serum concentration of galectin-1 was increased in BC patients in comparison with control group, however the difference was not statistically significant (*p* = 0.06; **Figure 1**).

**Figure 2:**
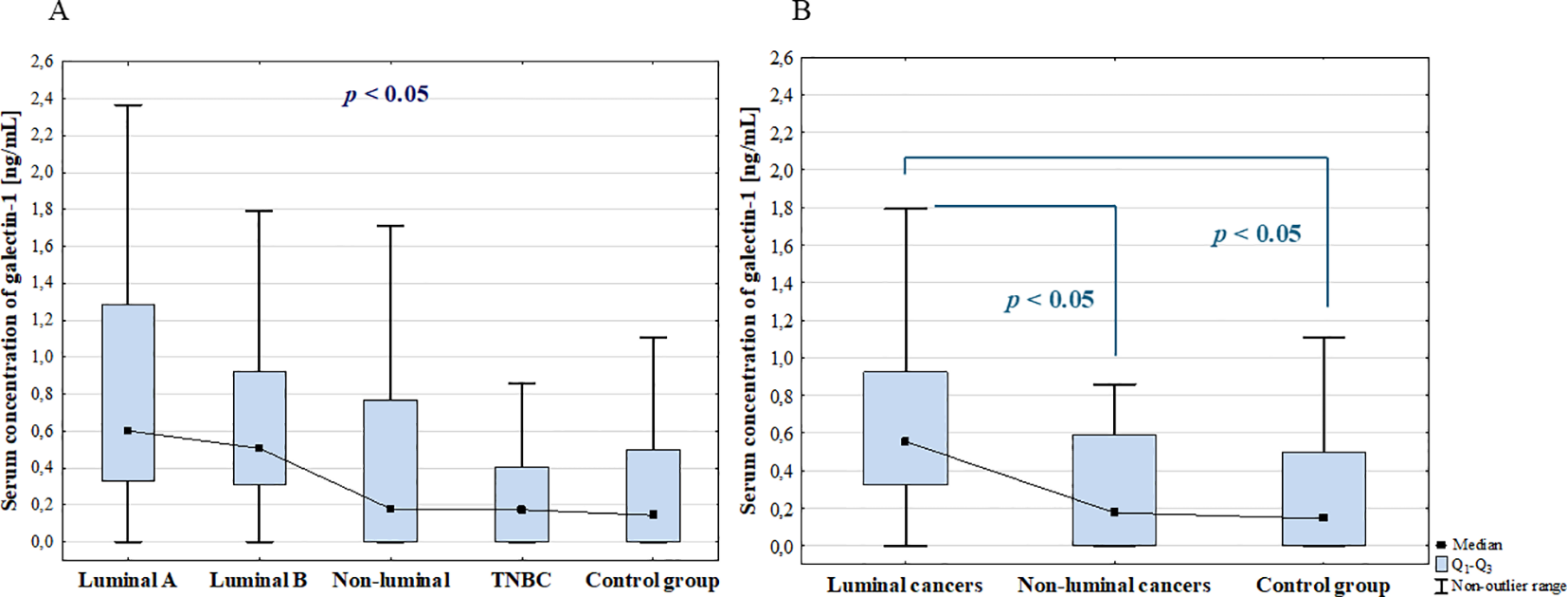
The comparison of serum concentration of galectin-1 among luminal A, luminal B, non-luminal HER2+, TNBC BC subtypes **(A)** and luminal (luminal A and luminal B) and non-luminal (non-luminal HER2+ and TNBC) cancers **(B)** and control group.

In the next step the serum concentrations of galectin-1 in BC patients with luminal A, luminal B, non-luminal HER2(+), TNBC and control group were compared. The ANOVA Kruskal-Wallis showed that there were statistically significant differences in the serum concentrations of galectin-1 among BC subgroups (*p* = 0.027), but *post-hoc* analysis did not reveal any statistically significant difference (**Figure 2A**). However, further analysis showed significantly higher concentration of galectin-1 in patients with luminal cancers (luminal A and luminal B) compared to non-luminal cancers (non-luminal HER2+ and TNBC) (*p* = 0.027) and control group (*p* = 0.031). No significant difference was found between non-luminal cancers (non-luminal HER2+ and TNBC) and control group (**Figure 2B**).

In additional analysis there were no statistically significant differences in serum galectin-1 concentrations among histological grades (G1, G2, G3) of BCs (*p* = 0.502) as well as between luminal B HER2 (–) and luminal B HER2(+) patients. Moreover, there was no significant difference between patients with and without metastasis (*p* = 0.848).

#### 3.1.2 Serum concentration of galectin-3

**Figure 3:**
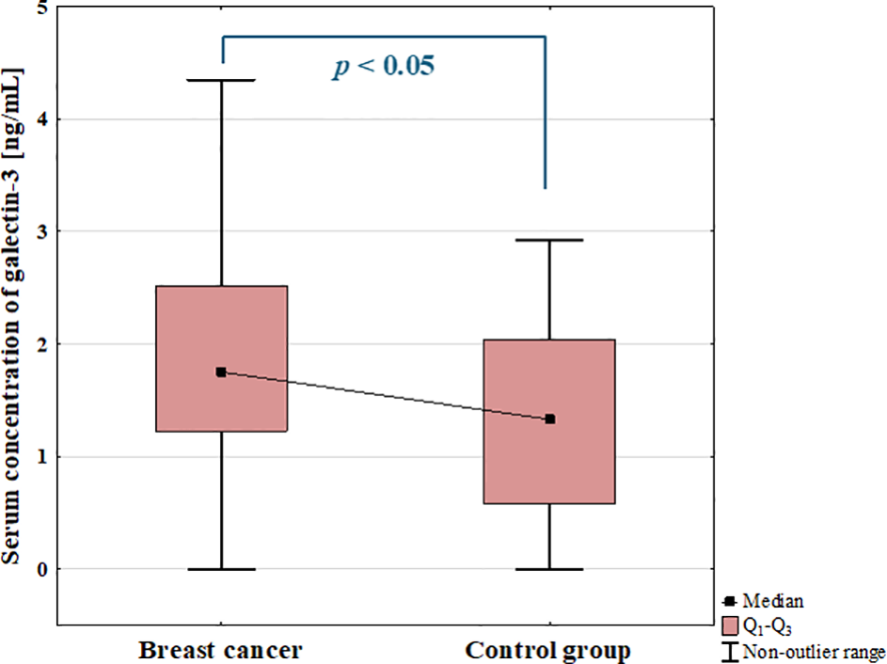
Serum concentration of galectin-3 in breast cancer patients and control group.

The results showed statistically significant increase in the serum concentration of galectin-3 in BC patients compared to control group (*p* = 0.029; **Figure 3**).

**Figure 4:**
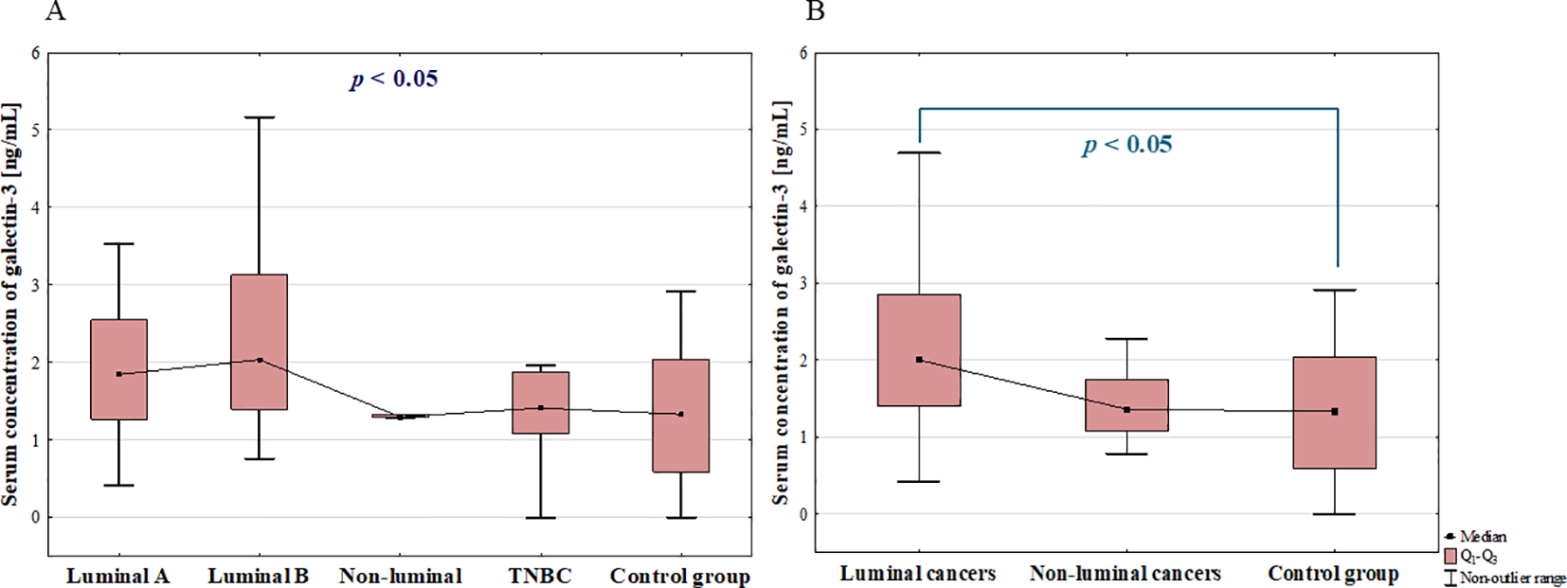
The comparison of serum concentration of galectin-3 among luminal A, luminal B, non-luminal HER2+, TNBC BC subtypes **(A)** and luminal (luminal A and luminal B) and non-luminal (non-luminal HER2+ and TNBC) cancers **(B)** and control group.

The further analysis revealed statistically significant differences in the serum concentration of galectin-3 among BC subtypes (*p* = 0.032), but *post-hoc* analysis did not show any significant difference between particular groups (**Figure 4A**). However, similarly as for galectin-1, the serum concentration of galectin-3 was significantly increased in luminal cancers compared to benign group (*p* = 0.017; **Figure 4B**).

Additionally, there were no statistically significant differences in serum galectin-3 concentrations among histological grades (G1, G2, G3) of BCs (*p* = 0.683) as well as between luminal B HER2 (–) and luminal B HER2(+) patients. Moreover, there was no significant difference between patients with and without metastasis (*p* = 0.249).

#### 3.1.3 Serum concentration of galectin-4

**Figure 5:**
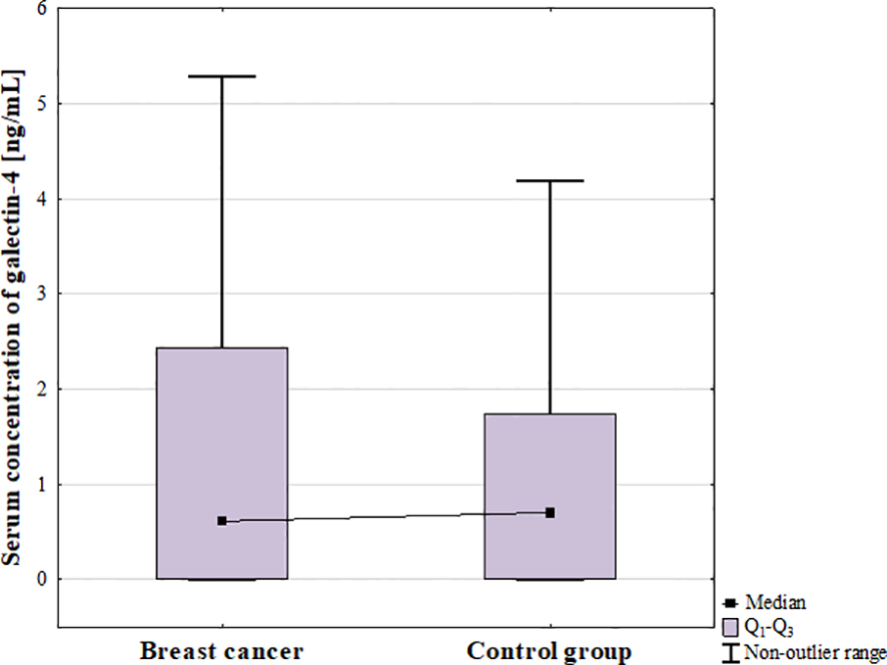
Serum concentration of galectin-4 in breast cancer patients and control group.

There was no statistically significant difference in serum concentration of galectin-4 between BC patients and control group (*p* = 1; **Figure 5**).

**Figure 6:**
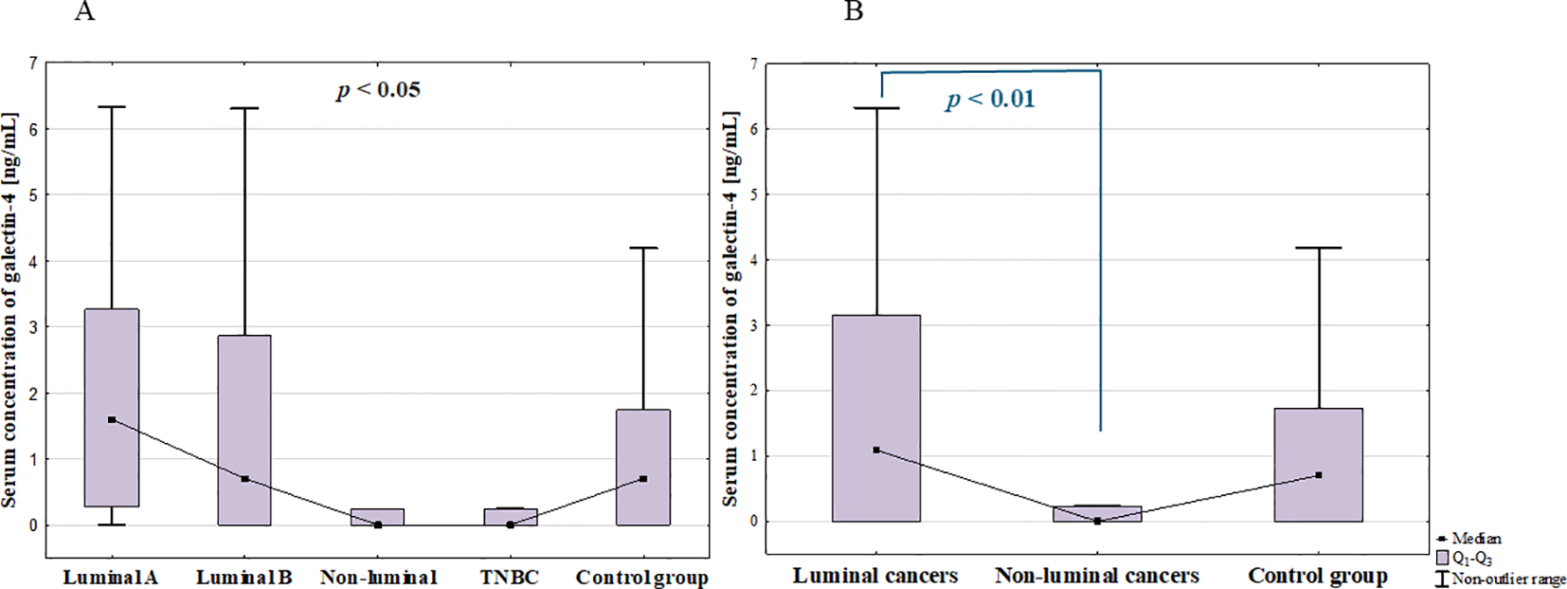
The comparison of serum concentration of galectin-4 among luminal A, luminal B, non-luminal HER2+, TNBC BC subtypes **(A)** and luminal (luminal A and luminal B) and non-luminal (non-luminal HER2+ and TNBC) cancers **(B)** and control group.

The analysis of the serum concentration of galectin-4 in BC subtypes showed significant differences among groups (*p* = 0.044; **Figure 6A**), however *post-hoc* analysis did not reveal any significant difference between groups. In further analysis, statistically significant difference between luminal and non-luminal cancers was found (*p* = 0.008; **Figure 6B**).

No significant differences were observed among histological grades (G1, G2, G3) of BCs (*p* = 0.5) as well as between luminal B HER2 (–) and luminal B HER2(+) patients. Moreover, there was no significant difference between patients with and without metastasis (*p* = 0.315).

#### 3.1.4 Serum concentration of galectin-7

**Figure 7:**
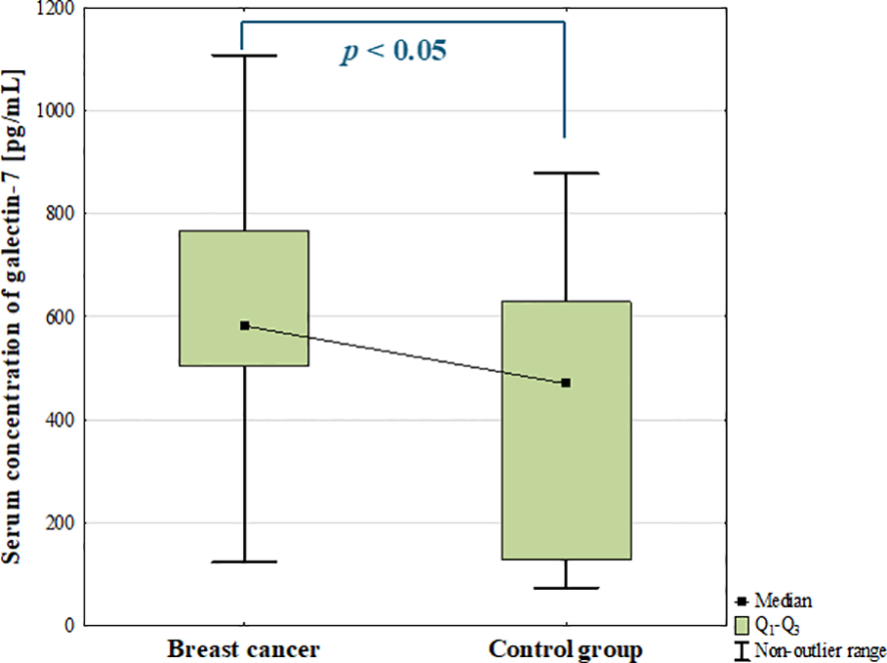
Serum concentration of galectin-7 in breast cancer and control group.

The serum concentration of galectin-7 was significantly increased in BC patients compared to the control group (*p* = 0.004; **Figure 7**).

**Figure 8:**
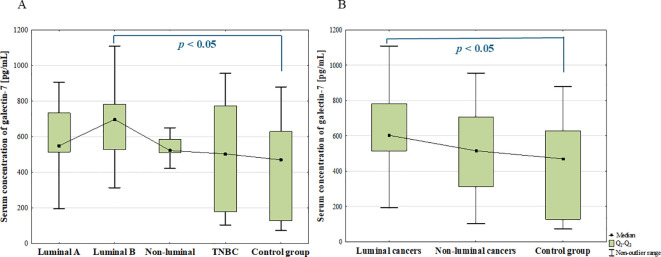
The comparison of serum concentration of galectin-7 among luminal A, luminal B, non-luminal HER2+, TNBC BC subtypes **(A)** and luminal (luminal A and luminal B) and non-luminal (non-luminal HER2+ and TNBC) cancers **(B)** and control group.

The ANOVA Kruskal-Wallis showed that there were statistically significant differences in the serum concentrations of galectin-7 among subtypes (*p* = 0.013). Additionally, *post-hoc* analysis revealed significantly increased serum concentration of galectin-7 in luminal B cancers compared to the control group (*p* = 0.006; **Figure 8A**). In patients with luminal tumours, significantly increased concentration of galectin-7 was also found compared to the control group (*p* = 0.003; **Figure 8B**).

Additionally, there were no statistically significant differences in serum galectin-7 concentrations among histological grades (G1, G2, G3) of BCs (*p* = 0.763) as well as between luminal B HER2 (–) and luminal B HER2(+) patients. Moreover, no significant difference was observed between patients with and without metastasis (*p* = 0.5).

#### 3.1.5 Serum concentration of galectin-9

**Figure 9:**
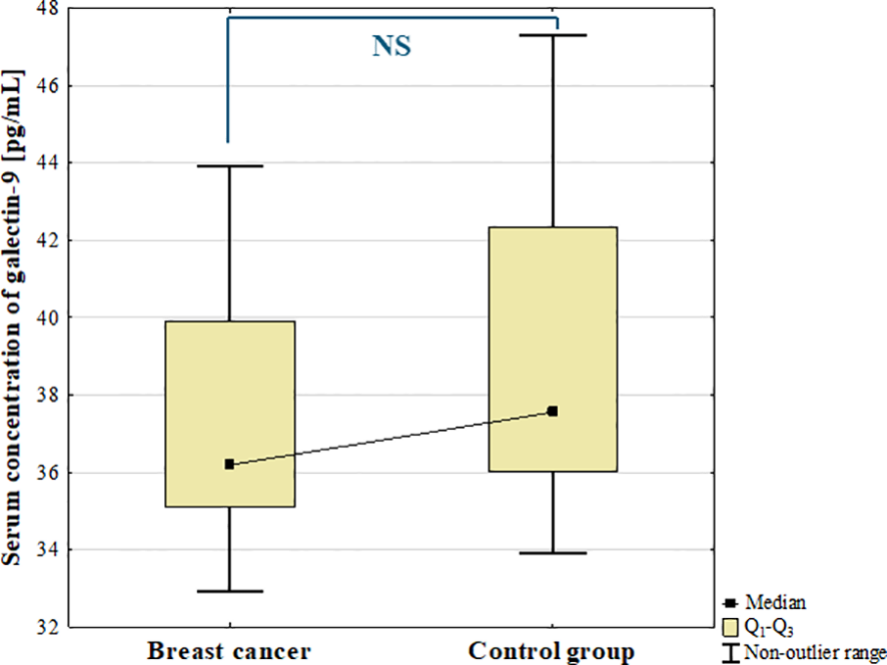
Serum concentration of galectin-9 in breast cancer and control group.

The results showed decreased concentration of galectin-9 in serum of BC patients compared to the control group. However, the difference was not statistically significant (*p* = 0.182; **Figure 9**).

Additionally, there were no statistically significant differences in the concentrations of galectin-9 among BC subtypes and control group (*p* = 0.489) nor among histological grades (G1, G2, G3) of BCs (*p* = 0.543) as well as between luminal B HER2 (–) and luminal B HER2(+) patients.

The next step was assessment of mRNA expression levels of genes encoding galectin-1 (*LGALS1*), -3 (*LGALS3*) and -7 (*LGALS7*). These genes were selected based on the results of protein concentration assessment for which statistically significant differences (galectin-3 and -7) or trends (galectin-1) were found.

### 3.2mRNA expression levels of galectins

#### 3.2.1 mRNA expression levels of galectin-1

**Figure 10:**
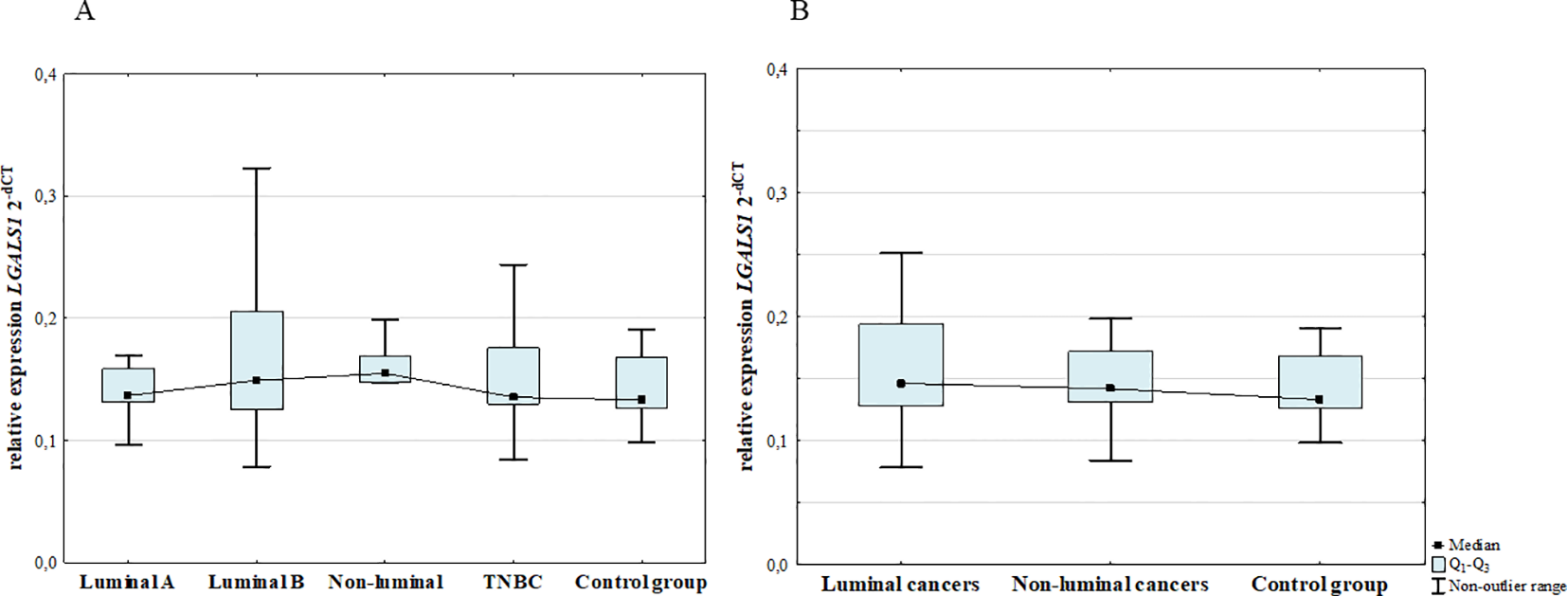
mRNA *LGALS1* expression among luminal A, luminal B, non-luminal HER2+, TNBC BC subtypes **(A)** and luminal (luminal A and luminal B) and non-luminal (non-luminal HER2+ and TNBC) cancers **(B)** and control group.

The mRNA of galectin-1 expression in the blood was present in all groups/samples. The analysis at the transcript level revealed no statistically significant differences in the expression of *LGALS1* mRNA level between BC patients and control group (*p* = 0.289). Moreover, there were no significant differences among BC subgroups (**Figure 10**).

#### 3.2.2 mRNA expression levels of galectin-3

The analysis at the transcript level did not show the expression of *LGALS3* mRNA level in BC patients and control group.

#### 3.2.3 mRNA expression levels of galectin-7

**Figure 11:**
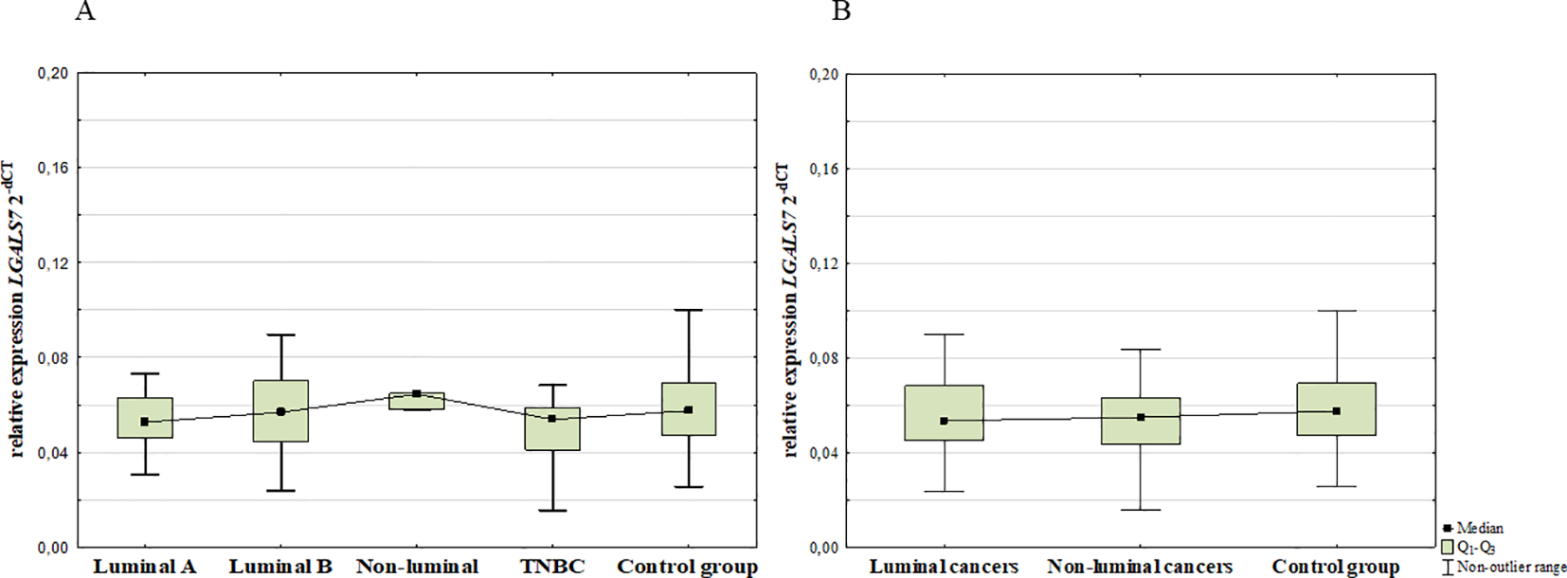
mRNA *LGALS7* expression among luminal A, luminal B, non-luminal HER2+, TNBC BC subtypes **(A)** and luminal (luminal A and luminal B) and non-luminal (non-luminal HER2+ and TNBC) cancers **(B)** and control group.

The mRNA of galectin-7 expression in the blood was present in all groups/samples. The analysis at the transcript level revealed no statistically significant differences in the expression of *LGALS7* mRNA level between BC patients and control group (*p* = 0.516). Moreover, there were no significant differences among BC subgroups (**Figure 11**).

### 3.3 Comparison with classical biomarkers

#### 3.3.1 Serum concentration of CA15-3

**Figure 12:**
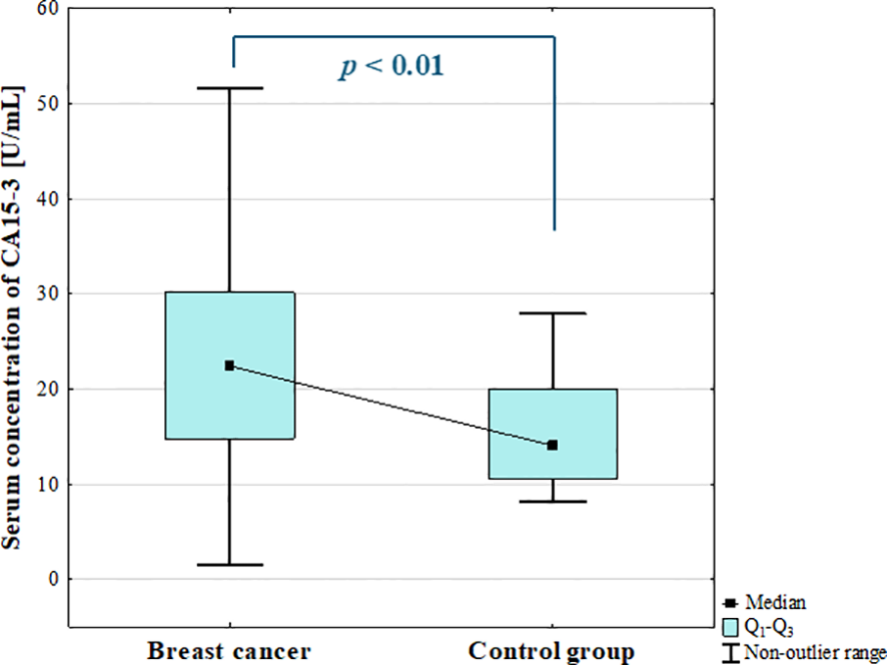
Serum concentration of CA15–3 in breast cancer and control group.

The analysis of the serum concentration of CA15–3 revealed significant increase in BC patients compared to the control group (*p* = 0.002; **Figure 12**).

**Figure 13:**
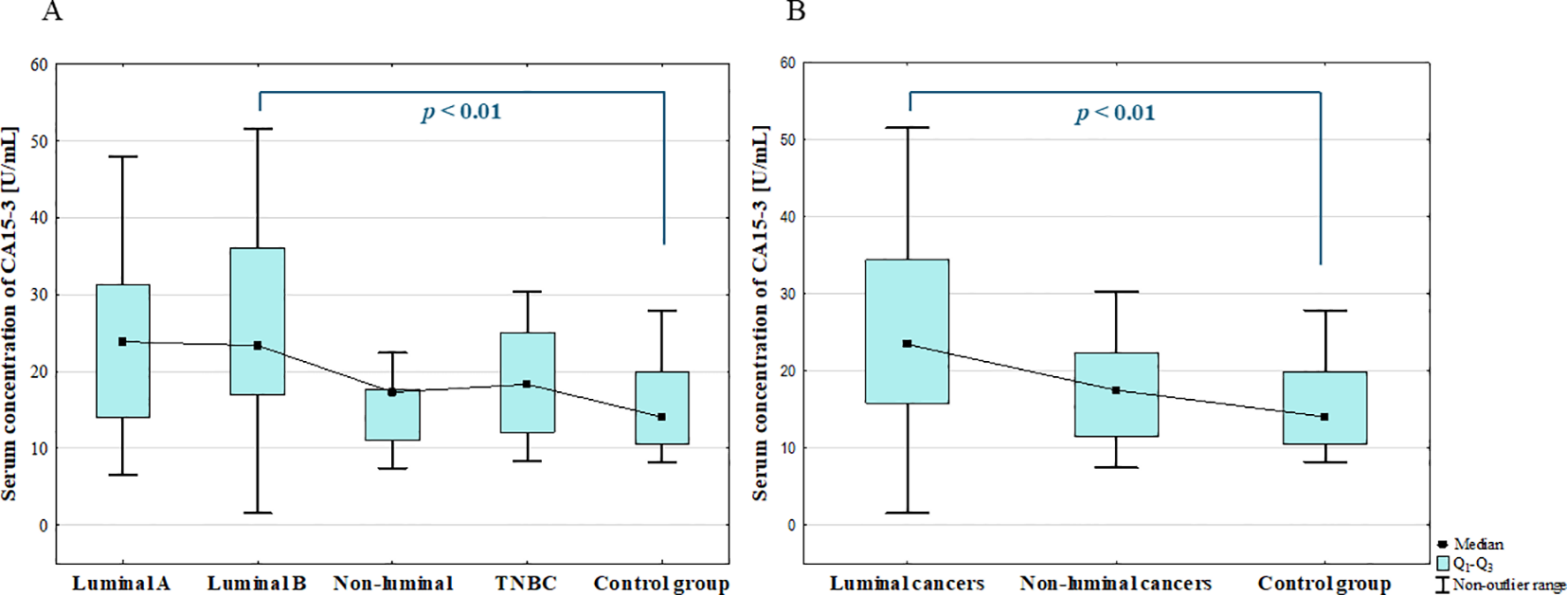
The comparison of the serum concentration of CA15–3 among luminal A, luminal B, non-luminal HER2+, TNBC BC subtypes **(A)** and luminal (luminal A and luminal B) and non-luminal (non-luminal HER2+ and TNBC) cancers **(B)** and control group.

The ANOVA Kruskal-Wallis showed that there were statistically significant differences in the serum concentrations of CA15–3 among BC subgroups (*p* = 0.005). Additionally, *post-hoc* analysis revealed significantly increased serum concentration of CA15–3 in luminal B cancers compared to benign group (*p* = 0.006; **Figure 13A**). Significantly increased concentration of CA15–3 in patients with luminal cancers compared to the control group (*p* = 0.001; **Figure 13B**) was also found.

Further analysis revealed significantly increased CA15–3 levels in BC patients with metastasis compared with patients without metastasis (*p* = 0.000). CA15–3 did not show any significant correlation with studied galectins.

#### 3.3.2 Serum concentration of CRP

**Figure 14:**
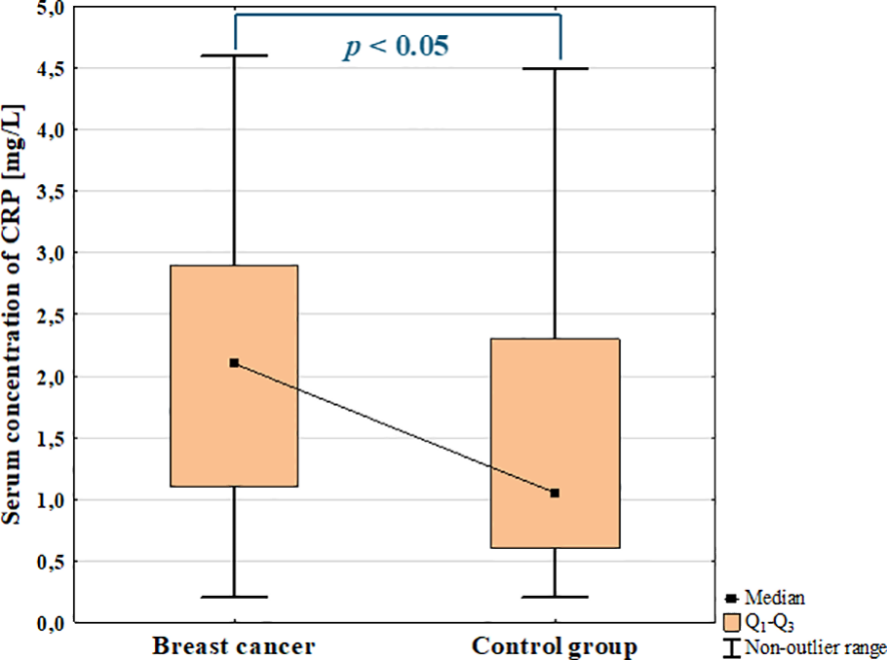
Serum concentration of CRP in breast cancer and control group.

The analysis of the serum concentration of CRP showed significant increase in BC patients compared to the control group (*p* = 0.028; **Figure 14**).

**Figure 15:**
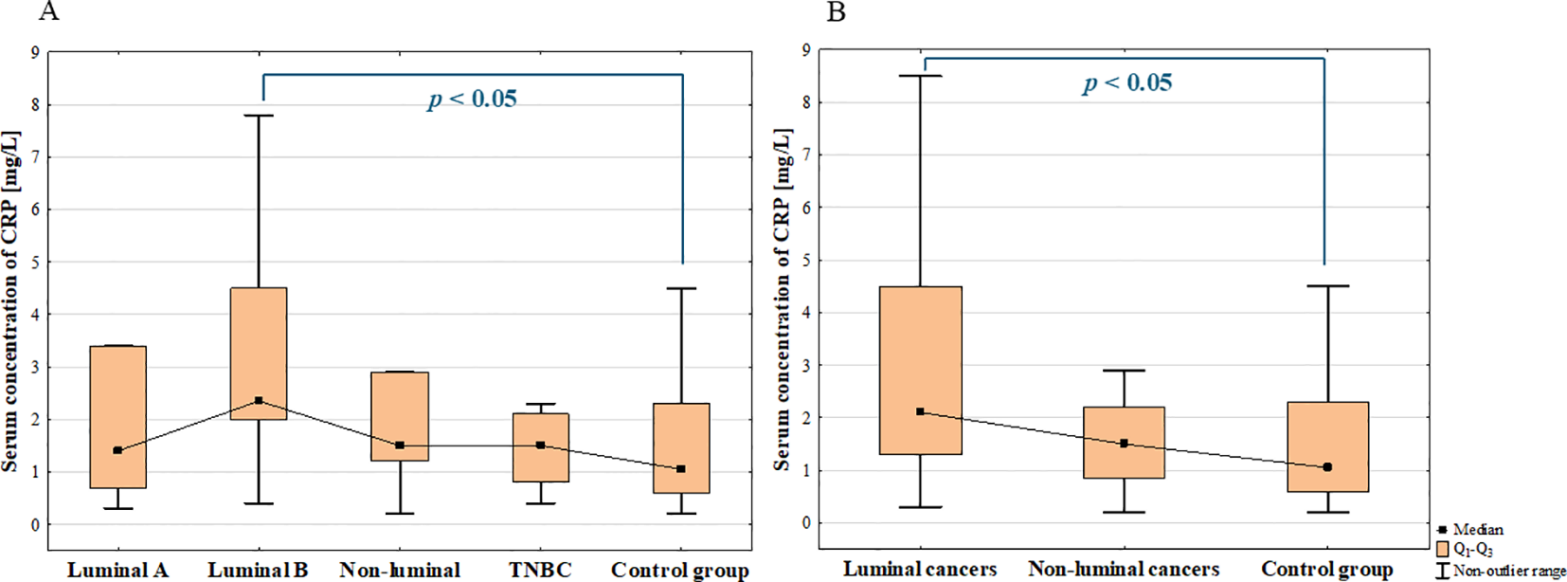
The comparison of the serum concentration of CA15–3 among luminal A, luminal B, non-luminal HER2+, TNBC BC subtypes **(A)** and luminal (luminal A and luminal B) and non-luminal (non-luminal HER2+ and TNBC) cancers **(B)** and control group.

The ANOVA Kruskal-Wallis showed that there were statistically significant differences in the serum concentrations of CRP among groups (*p* = 0.021). Additionally, *post-hoc* analysis revealed significantly increased serum concentration of CRP in luminal B cancers compared to the control group (*p* = 0.017; **Figure 15A**). Increased concentration of CRP was observed in patients with luminal cancers compared with control group (*p* = 0.032; **Figure 15B**) as well as in patients with metastasis compared to non-metastasis (*p* = 0.003).

**Figure 16:**
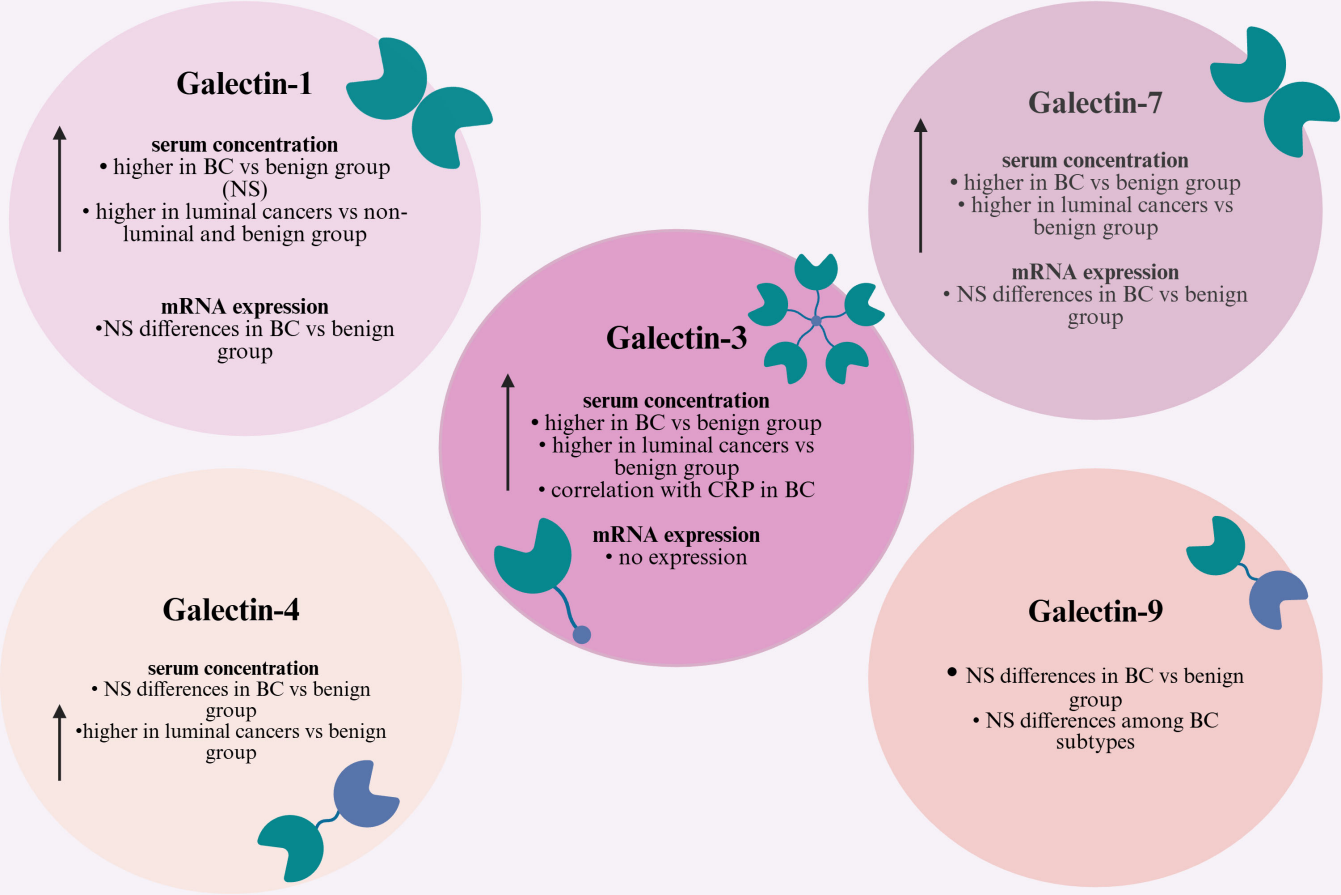
Summary of the obtained results for the concentration and mRNA expression of galectins in breast cancer (Created in BioRender. Mielczarek-Palacz, A. (2026) https://BioRender.com/5fq4ju0).

Additionally, a weak positive correlation was found between serum concentrations of CRP and galectin-3 in BC patients (rS = 0,27, *p* = 0.04). The summary of the obtained results is presented in **Figure 16**.

### 3.4 Diagnostic performance

#### 3.4.1 ROC curve analysis

**Figure 17:**
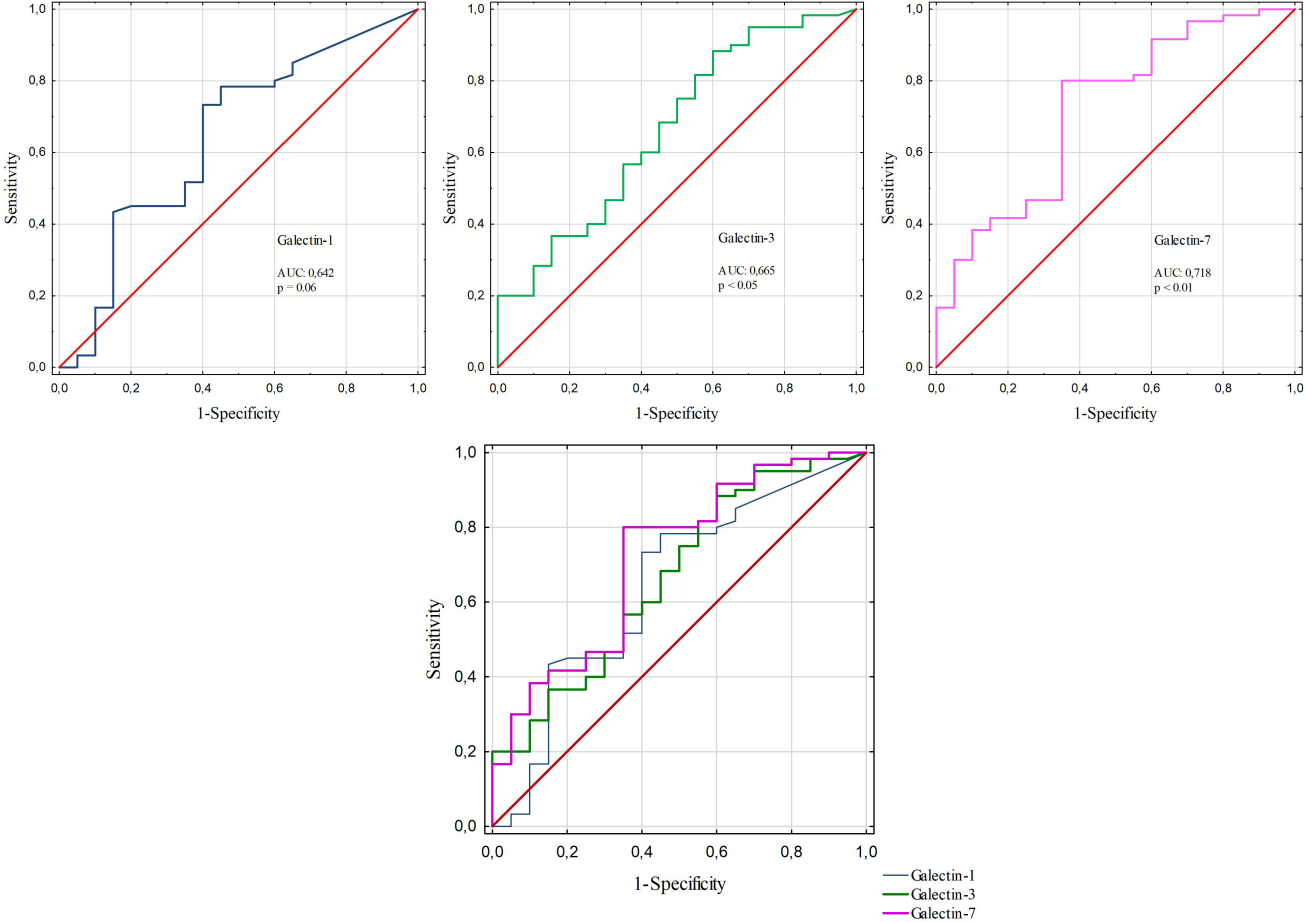
ROC curve for serum galectin-1, -3 and -7.

ROC curve analysis was performed to evaluate the diagnostic value of galectin-1, -3, and -7, CA15–3 and CRP as well as combinations of studied galectins with classic markers in distinguishing BC from benign breast disease. Among galectins analysed in this study the best diagnostic value performed galectin-7 (AUC: 0,718; **Figure 17**). However, for combinations the best results were achieved for galectin-3. The best diagnostic value performed a combination of galectin-3 and CA15–3 with AUC 0,742 and a combination of galectin-3 and CA15–3 and CRP with AUC 0,754 (**Figure 16**; **Table 5**).

**Table 5:** The diagnostic value of studied parameters and their combinations in discrimination breast cancer from benign breast disease.

Parameter	AUC (95% CI)	Sensitivity [%]	Specificity [%]	*p*
Galectin-1	0,642 (0,494-0,79)	78,3	55,0	0.0609
Galectin-3	0,665 (0,525-0,804)	88,3	40	0.0208*
Galectin-7	0,718 (0,585-0,851)	80,0	65,0	0.0013*
CA15-3	0,732 (0,62-0,843)	51,7	95,0	0.0000*
CRP	0,665 (0,527-0,804)	71,2	60	0.0194*
Gal-1 + CA15-3	0,738 (0,628-0,848)	53,3	95,0	0.0000*
Gal-1 + CA15-3 + CRP	0,744 (0,63-0,858)	59,3	85,0	0.0000*
Gal-3 + CA15-3	0,742 (0,632-0,852)	51,7	95,0	0.0000*
Gal-3 + CA15-3 + CRP	0,754 (0,644-0,865)	69,5	75,0	0.0000*
Gal7 + CA15-3	0,728 (0,597-0,86)	81,7	60,0	0.0007*
Gal7 + CA15-3 + CRP	0,734 (0,603-0,865)	83,1	60	0.0005*

CI , confidence interval; *p < 0.05 statistically significant.

Conclusively, the findings indicate that circulating galectins, particularly galectin-3 and galectin-7, are elevated in patients with invasive BC, especially within luminal molecular subtypes. Their combinations with established markers such as CA15–3 and CRP may improve diagnostic performance.

## 4 Discussion

### 4.1 Interpretation of the results in the context of previous studies

The significance of galectins in various cancers, including BC, has been extensively studied at both protein and molecular levels. This study focussed on the analysis of galectins in the blood of women with BC and benign breast disease including serum concentrations and mRNA expression. The results of this analysis revealed increased serum concentrations of galectin-1, -3 and -7 in BC patients compared to the benign breast group, however mRNA expression of these galectins did not show any significant differences between these groups of women. In the study group, especially women with luminal cancers higher serum concentrations of galectin-1, -3 and -7 was observed compared to the control group.

Similar results were obtained by Blair et al. who found increased serum concentrations of galectin-1, -3 and -7 in BC patients compared to healthy controls (17). Funkhouser et al. showed higher serum levels of galectin-1, -3, -8 and -9 in BC patients with a mutation in the *KIT* gene (18). Moreover, mutations in this gene were more likely to be found in tissue samples of patients with brain metastases (18). In this analysis no differences have been found in the serum concentrations of galectins between patients with and without metastasis. Similar results for galectin-3 were observed by Topcu et al. who found increased serum levels of galectin-3 in BC patients compared to healthy controls (19). Moreover, they also showed no significant differences in galectin-3 serum levels in BC patients with and without metastasis (19).

Contrary to the results of this analysis, Barrow et al. found increased serum concentrations of galectin-2, -4 and -8, and decreased concentration of galectin-1 in BC patients compared to healthy controls (20). In this study, no serum concentrations of galectin-2 and -8 were detectable either in BC and benign breast lesions patients. Moreover, serum concentration of galectin-1 was increased in BC patients, while concentration of galectin-4 in BC patients was not significantly different from benign breast lesions. In another study by Funkhouser et al. the authors found significantly increased serum levels of galectin-9 in HER2-enriched tumours whereas decreased levels were observed in patients with hormone-receptor-positive tumours (21). This study revealed decreased serum levels of galectin-9 in BC patients compared to benign group, however this difference was not statistically significant and there were no differences among BC molecular subtypes.

In this study, the most promising results of serum concentration analysis were obtained for galectin-1, -3 and -7. Therefore, we decided to analyse mRNA expression levels of *LGALS1*, *LGALS3* and *LGALS7* in the blood of BC patients and women with benign breast disease. To the best of our knowledge, this is the first study assessing both circulating and transcriptional galectin profiles in breast cancer. The results showed no significant changes in *LGALS1* and *LGALS7* mRNA expression between BC patients and the control group. It should be emphasised that the expression level of *LGALS1* was significantly higher than that of *LGALS7*. Moreover, we did not find the expression of *LGALS3* mRNA in both BC patients and the control group. These discrepancies suggest that serum galectins 1, 3 and 7 originate from different sources. In the case of galectin-1, low serum concentration (0,45 ng/ml in invasive BC and 0,06 ng/ml in control group) and high mRNA expression level may indicate that circulating immune cells may constitute a significant source of its expression. The lack of statistical difference in both protein concentration (only a trend was observed) and mRNA expression level seems to confirm this assumption, but further studies are required. In the case of galectin-7 high protein concentration in serum was found (582.16 ng/ml in invasive BC and 469.60 ng/ml in control group) and low mRNA expression level in circulating immune cells. Moreover, protein concentration showed a statistically significant difference, unlike mRNA expression level, which may indicate that galectin-7 is derived mainly from tumour or stromal secretion rather than circulating immune cells. In the case of galectin-3, protein concentration in the serum was quite low (1.75 ng/ml in invasive BC and 1.33 ng/ml in control group) and there was a lack of mRNA expression, which may indicate that this lectin originates from tumour or stromal secretion. Given these findings and the fact that the combination of galectin-3 and CA15–3 and CRP presents the best diagnostic value of (AUC 0,754), galectin-3 may constitute a parameter independent of factors present in the circulation, but this requires further in-depth research.

The discrepancy between serum galectin protein levels and whole blood mRNA expression likely reflects differences in cellular origin and regulatory mechanisms rather than contradictory findings. Circulating galectins may derive predominantly from tumour cells, stromal cells, or endothelial cells, whereas mRNA extracted from whole blood primarily represents leukocyte expression. Additionally, post-transcriptional regulation, protein stability, and secretion dynamics may further contribute to the observed differences. These mechanisms could explain why galectin-3 mRNA was undetectable in blood and why LGALS1 and LGALS7 transcripts did not differ between BC patients and controls, despite significant elevations in their corresponding serum proteins. Recognising the compartment-specific regulation of galectin expression provides important context for interpreting circulating galectin levels as potential biomarkers in breast cancer.

### 4.2 Potential biological mechanisms and diagnostic implications

Galectins are suggested to play a role in cancer-associated inflammation contributing to tumour progression and metastasis (22). Galectin-1 is known to be secreted by tumour stroma, tumour-infiltrated immune cells and tumour cells contributing to immune evasion and cancer progression. Its immunomodulatory effects within tumour microenvironment (TME), which facilitates tumour immune escape, include promoting the apoptosis of effector T cells, inhibiting NK cells function or promoting macrophage change from M1 to M2 phenotype (23–25). It was observed that serum levels of galectin-1 decreased in postoperative BC patients compared to preoperative concentrations what suggest its importance in carcinogenesis and positive correlation with the presence of tumour (26).

Depending on BC molecular subtype, galectin-1 was reported to regulate different cancer progression pathways and its knockdown was found to inhibit cancer progression in hormone-receptor positive BC cells by regulating cell-cycle whereas in TNBC cells by regulating epithelial-mesenchymal transition (EMT) mechanisms (27). In breast tumour models Chung et al. revealed that inhibition of galectin-1 reinvigorates dysfunctional CD8+ T cells causing antitumour immune response in TME (28). Suppressing galectin-1 expression was also found to reduce tumour growth and lung metastases in BC model (29).

Galectin-3 is present in the nucleus and cytoplasm, but also on the cell surface and it can be secreted to extracellular matrix (ECM) (13). It plays a role in diverse biological activities, for example in cell growth, apoptosis, angiogenesis but also in inflammation and fibrosis (30, 31). It is suggested that galectin-3 promotes tumour progression and metastasis through different mechanisms including the inhibition of cancer cells apoptosis by interactions between cytoplasmic galectin-3 with Bcl-2 (30, 32).

In TNBC galectin-3 was found to perform an immunosuppressive mechanism that affects CD4 and CD8 cells (33). Galectin-3 secreted by TNBC cells induced an increase in tumour infiltrating T-regulatory cells and secretion of the higher levels of immunosuppressive cytokines IL-10 and IL-35. Moreover, this lectin was shown to induce mitochondrial dysfunction that can led to increased intracellular ROS production in CD8 cells that results in an increase in T-exhausted cells (33). Galectin-3 was found to be a marker of chemotherapy efficacy in BC patients as well as it was shown to be associated with high tumour size in BC patients without response to chemotherapy (34, 35). Zheng et al. demonstrated that secreted by tumour cells galectin-3 promotes IL-10 production in B cells impairing antitumour immune response (36). Due to the ability of galectin-3 to modulate immune response it is considered to be a potential target molecule to overcome tumour immune escape and improve the efficacy of chemotherapy in BC (36, 37).

The immunomodulatory mechanisms of galectin-7 in cancers are less studied than those of galectin-1 and -3. However, recent findings reported its involvement in various cancers, including breast cancer. Galectin-7 was found to promote the invasiveness of oral squamous cell carcinoma cells by inducing the expression of protein matrix metalloproteinase-2 (MMP-2) and MMP-9 through activation of ERK and JNK signalling (38). High expression levels of galectin-7 in breast carcinoma tissue were shown to be associated with aggressive BC subtypes, including HER2 overexpressing and basal-like, and with increased metastasise ability of BC cells (39). It was also reported that galectin-7 can potentiate HER-2 positive phenotype in primary BC (40). Trebo et al. demonstrated that high galectin-7 expression in the cytoplasm was a negative prognostic factor for survival in BC patients, while high galectin-8 expression was associated with improved patients outcome (41).

In recent study, galectin-7 was demonstrated to bind to PD-1, which leads to the suppression of TCR pathway and contributes to the reduction of CD4+ T cells in tumour microenvironment (42). In BC cells galectin-7 was found to induce chemoresistance by its interactions with p53, which impair the translocation of p53 to the nucleus (43). Promising results were obtained by Nehmè et al., who used nanobodies to develop galectin-7-specific inhibitors, which may open new possibilities for targeted therapy of cancers, including TNBC (44).

The observed differences between circulating protein levels and mRNA expression in whole blood suggest that galectin regulation is complex and compartment-specific. Circulating galectins likely originate from multiple cellular sources, including tumour, stromal, and endothelial cells, rather than blood leukocytes alone. Moreover, post-transcriptional regulation, protein secretion dynamics, and differential protein stability can contribute to elevated serum levels independently of mRNA abundance in circulating cells. These mechanisms may explain why LGALS3 transcripts were undetectable in blood, and why LGALS1 and LGALS7 mRNA levels did not significantly differ between BC patients and controls, despite substantial increases in serum protein concentrations. Considering these processes is crucial when interpreting galectin levels as potential biomarkers or therapeutic targets in breast cancer.

### 4.3 Clinical relevance and correlations with classical markers

In this study BC patients had significantly increased concentrations of CA15–3 and CRP which was especially observed in women with luminal B cancers. Similarly, serum concentrations of galectin-1, -3 and -7 were particularly increased in luminal cancers. Moreover, galectin-3 was found to correlate with the levels of CRP in BC patients. All these findings may indicate the role of galectins in inflammation associated with cancer.

Among combinations of markers the most promising results were obtained for combinations of classic markers CA15–3 and CRP with galectin-3 (AUC: 0,754), galectin-1 (AUC: 0,744) and galectin-7 (AUC: 0,734). The addition of these galectins improved the discriminating value of classical markers. These results may suggest the potential role of galectins as diagnostic biomarkers in BC patients. However, additional analyses including external validation, cross-validation or comparison with standard diagnostic workflows in a larger independent cohort are required. Therefore, our findings indicating the diagnostic utility of galectins remain preliminary.

### 4.4 Limitations and future perspectives

A limitation of our analysis is relatively small group of patients with the most aggressive BC subtypes including non-luminal HER2+ and TNBC, which inevitably limits statistical power and weakens the robustness of subtype comparisons. Therefore, the subtype-specific findings should be interpreted with caution and considered exploratory. Moreover, as this study is the first that assesses circulating and transcriptional galectin profile across BC molecular subtypes the further analyses with larger numbers of patients are required, especially for the most aggressive subtypes, which are generally less common than luminal cancers.

Another important limitation of the present study is the significant age difference between breast cancer patients and the control group, as well as the use of patients with benign breast lesions as controls. Since circulating inflammatory markers, including galectins and CRP, may be influenced by age-related immune changes and inflammatory processes associated with benign breast conditions, these factors may represent potential confounders and could partially affect the interpretation of the observed differences. Due to the limited size of the control group, age-matched analyses or robust multivariable adjustment were not feasible without compromising statistical power. Therefore, the findings of this study should be considered exploratory and warrant validation in larger, age-matched cohorts, including healthy controls, to better delineate cancer-specific alterations in circulating galectins.

The method of determining serum concentrations of galectins used in this study has also limitations. Reliable interpretation of ELISA results is only possible within the detection range for a given test and is limited by the sensitivity of the assay. Therefore, the results below the detection range does not exclude the presence of the analyte. Due to the comparative nature of this analysis, which concerned the determination of serum concentrations of parameters related to the inflammatory process, it was decided not to exclude the results below and above the detection range in order not to lose important data. It should be emphasise, however, that this approach may potentially cause bias.

This study highlighted the role of galectins in cancer immune response and their potential use as serum biomarkers to improve diagnostic performance in BC. The importance of galectins in diagnosis, prognosis and targeted therapy has been widely studied, and numerous clinical trials have explored their therapeutic potential across various cancers (13, 45). A thorough understanding of mechanisms by which galectins can influence development and progression of cancer may be essential in improving early diagnosis, effective therapy and survival of BC patients.

## 5 Conclusions

1. The results obtained suggest that increased serum concentrations of galectins, especially galectin-3 and -7, may result from their release by breast cancer cells into the bloodstream, which may indicate their potential importance in tumour development.
2. The determination of galectin-1, -3 and -7 with classic markers CA15–3 and CRP may prove helpful in the differential diagnosis of patients with breast cancer and benign lesions as well as may confirm the importance of these lectins in the development of inflammatory reactions, having potential usefulness in monitoring the inflammatory process in the course of the disease.
3. The demonstrated presence of galectins derived from cancer cells in the blood of women with breast cancer provides a basis for further research into their use as potential biomarkers, useful both in diagnosis and monitoring of the course of the disease and also creates opportunities for application in modern targeted therapy.

## Data Availability

The raw data supporting the conclusions of this article will be made available by the authors, without undue reservation.

## References

[1] Bray F Laversanne M Sung H Ferlay J Siegel RL Soerjomataram I . Global cancer statistics 2022: GLOBOCAN estimates of incidence and mortality worldwide for 36 cancers in 185 countries. CA Cancer J Clin. (2024) 74:229–63. doi: 10.3322/caac.21834, PMID: 38572751

[2] Wilkinson L Gathani T . Understanding breast cancer as a global health concern. Br J Radiol. (2022) 95:20211033. doi: 10.1259/bjr.20211033, PMID: 34905391 PMC8822551

[3] Barba D León-Sosa A Lugo P Suquillo D Torres F Surre F . Breast cancer, screening and diagnostic tools: All you need to know. Crit Rev Oncol Hematol. (2021) 157:103174. doi: 10.1016/j.critrevonc.2020.103174, PMID: 33249359

[4] Burciu OM Sas I Popoiu T-A Merce A-G Moleriu L Cobec IM . Correlations of imaging and therapy in breast cancer based on molecular patterns: an important issue in the diagnosis of breast cancer. Int J Mol Sci. (2024) 25:8506. doi: 10.3390/ijms25158506, PMID: 39126074 PMC11312504

[5] Xiong X Zheng L-W Ding Y Chen Y-F Cai Y-W Wang L-P . Breast cancer: pathogenesis and treatments. Signal Transduct Target Ther. (2025) 10:49. doi: 10.1038/s41392-024-02108-4, PMID: 39966355 PMC11836418

[6] Vasconcelos I Hussainzada A Berger S Fietze E Linke J Siedentopf F . The St. Gallen surrogate classification for breast cancer subtypes successfully predicts tumor presenting features, nodal involvement, recurrence patterns and disease free survival. . Breast. (2016) 29:181–5. doi: 10.1016/j.breast.2016.07.016, PMID: 27544822

[7] Sarhangi N Hajjari S Heydari SF Ganjizadeh M Rouhollah F Hasanzad M . Breast cancer in the era of precision medicine. Mol Biol Rep. (2022) 49:10023–37. doi: 10.1007/s11033-022-07571-2, PMID: 35733061

[8] Orrantia-Borunda E Anchondo-Nuñez P Acuña-Aguilar LE Gómez-Valles FO Ramírez-Valdespino CA . Subtypes of breast cancer. In: Mayrovitz HN , editor. Breast cancer. Exon Publications, Brisbane (AU (2022). Available online at: http://www.ncbi.nlm.nih.gov/books/NBK583808/. 36122153

[9] Barondes SH Cooper DN Gitt MA Galectins LH . Structure and function of a large family of animal lectins. . J Biol Chem. (1994) 269:20807–10. doi: 10.1016/S0021-9258(17)31891-4 8063692

[10] Yang R-Y Rabinovich GA Liu F-T . Galectins: structure, function and therapeutic potential. Expert Rev Mol Med. (2008) 10:e17. doi: 10.1017/S1462399408000719, PMID: 18549522

[11] Vasta GR . Galectins as pattern recognition receptors: structure, function, and evolution. Adv Exp Med Biol. (2012) 946:21–36. doi: 10.1007/978-1-4614-0106-3_2, PMID: 21948360 PMC3429938

[12] Fuselier C Dumoulin A Paré A Nehmé R Ajarrag S Granger Joly de Boissel P . Placental galectins in cancer: why we should pay more attention. Cells. (2023) 12:437. doi: 10.3390/cells12030437, PMID: 36766779 PMC9914345

[13] Zhang N Liu Q Wang D Wang X Pan Z Han B . Multifaceted roles of Galectins: from carbohydrate binding to targeted cancer therapy. biomark Res. (2025) 13:49. doi: 10.1186/s40364-025-00759-1, PMID: 40134029 PMC11934519

[14] Abdelfattah MM Helwa R . Galectins dysregulation: A way for cancer cells to invade and pervade. Oncol Res. (2023) 30:129–35. doi: 10.32604/or.2022.026838, PMID: 37305017 PMC10208010

[15] Chou F-C Chen H-Y Kuo C-C Sytwu H-K . Role of galectins in tumors and in clinical immunotherapy. Int J Mol Sci. (2018) 19:430. doi: 10.3390/ijms19020430, PMID: 29389859 PMC5855652

[16] Kapetanakis N-I Busson P . Galectins as pivotal components in oncogenesis and immune exclusion in human Malignancies. Front Immunol. (2023) 14:1145268. doi: 10.3389/fimmu.2023.1145268, PMID: 36817445 PMC9935586

[17] Blair BB Funkhouser AT Goodwin JL Strigenz AM Chaballout BH Martin JC . Increased circulating levels of galectin proteins in patients with breast, colon, and lung cancer. Cancers (Basel). (2021) 13:4819. doi: 10.3390/cancers13194819, PMID: 34638303 PMC8508020

[18] Funkhouser AT Strigenz AM Blair BB Miller AP Shealy JC Ewing JA . KIT mutations correlate with higher galectin levels and brain metastasis in breast and non-small cell lung cancer. Cancers (Basel). (2022) 14:2781. doi: 10.3390/cancers14112781, PMID: 35681762 PMC9179545

[19] Topcu TO Kavgaci H Gunaldi M Kocoglu H Akyol M Mentese A . The clinical importance of serum galectin-3 levels in breast cancer patients with and without metastasis. J Cancer Res Ther. (2018) 14:S583–6. doi: 10.4103/0973-1482.176425, PMID: 30249872

[20] Barrow H Guo X Wandall HH Pedersen JW Fu B Zhao Q . Serum galectin-2, -4, and -8 are greatly increased in colon and breast cancer patients and promote cancer cell adhesion to blood vascular endothelium. Clin Cancer Res. (2011) 17:7035–46. doi: 10.1158/1078-0432.CCR-11-1462, PMID: 21933892

[21] Funkhouser A Shuster H Martin JC Edenfield WJ Blenda AV . Pattern analysis of serum galectins-1, -3, and -9 in breast cancer. Cancers (Basel). (2023) 15:3809. doi: 10.3390/cancers15153809, PMID: 37568625 PMC10417135

[22] Kruk L Braun A Cosset E Gudermann T Mammadova-Bach E . Galectin functions in cancer-associated inflammation and thrombosis. Front Cardiovasc Med. (2023) 10:1052959. doi: 10.3389/fcvm.2023.1052959, PMID: 36873388 PMC9981828

[23] Novák J Takács T Tilajka Á László L Oravecz O Farkas E . The sweet and the bitter sides of galectin-1 in immunity: its role in immune cell functions, apoptosis, and immunotherapies for cancer with a focus on T cells. Semin Immunopathol. (2025) 47:24. doi: 10.1007/s00281-025-01047-8, PMID: 40178639 PMC11968517

[24] Griffiths A Udomjarumanee P Georgescu A-S Barri M Zinovkin DA Pranjol MZI . The immunomodulatory role of galectin-1 in the tumour microenvironment and strategies for therapeutic applications. Cancers (Basel). (2025) 17:1888. doi: 10.3390/cancers17111888, PMID: 40507367 PMC12153884

[25] Huang Y Wang H-C Zhao J Wu M-H Shih T-C . Immunosuppressive roles of galectin-1 in the tumor microenvironment. Biomolecules. (2021) 11:1398. doi: 10.3390/biom11101398, PMID: 34680031 PMC8533562

[26] Gurel Cayir E Demir L Varol U Atahan MK Salman T Oflazoglu U . Preliminary study of serum Galectin-1 in breast cancer carcinogenesis [Izmir Oncology Group (IZOG) study. J BUON. (2020) 25:675–80. 32521852

[27] Kim JY Lee JH Jung EJ Son YS Park HJ Kim JM . Therapeutic targeting of the galectin-1/miR-22-3p axis regulates cell cycle and EMT depending on the molecular subtype of breast cancer. Cells. (2025) 14:310. doi: 10.3390/cells14040310, PMID: 39996781 PMC11854374

[28] Chung H Gyu-Mi P Na YR Lee Y-S Choi H Seok SH . Comprehensive characterization of early-programmed tumor microenvironment by tumor-associated macrophages reveals galectin-1 as an immune modulatory target in breast cancer. Theranostics. (2024) 14:843–60. doi: 10.7150/thno.88917, PMID: 38169569 PMC10758049

[29] Dalotto-Moreno T Croci DO Cerliani JP Martinez-Allo VC Dergan-Dylon S Méndez-Huergo SP . Targeting galectin-1 overcomes breast cancer-associated immunosuppression and prevents metastatic disease. Cancer Res. (2013) 73:1107–17. doi: 10.1158/0008-5472.CAN-12-2418, PMID: 23204230

[30] Ahmed R Anam K Ahmed H . Development of galectin-3 targeting drugs for therapeutic applications in various diseases. Int J Mol Sci. (2023) 24:8116. doi: 10.3390/ijms24098116, PMID: 37175823 PMC10179732

[31] Dong R Zhang M Hu Q Zheng S Soh A Zheng Y . Galectin-3 as a novel biomarker for disease diagnosis and a target for therapy (Review). Int J Mol Med. (2018) 41:599–614. doi: 10.3892/ijmm.2017.3311, PMID: 29207027 PMC5752178

[32] Newlaczyl AU Yu L-G . Galectin-3--a jack-of-all-trades in cancer. Cancer Lett. (2011) 313:123–8. doi: 10.1016/j.canlet.2011.09.003, PMID: 21974805

[33] Raiter A Barhum Y Lipovetsky J Menachem C Elgavish S Ruppo S . Galectin-3 secreted by triple-negative breast cancer cells regulates T cell function. Neoplasia. (2025) 60:101117. doi: 10.1016/j.neo.2024.101117, PMID: 39729650 PMC11742317

[34] Shafiq A Moore J Suleman A Faiz S Farooq O Arshad A . Elevated soluble galectin-3 as a marker of chemotherapy efficacy in breast cancer patients: A prospective study. Int J Breast Cancer. (2020) 2020:4824813. doi: 10.1155/2020/4824813, PMID: 32231800 PMC7097759

[35] Niang DGM Ka S Hendricks J Diouf D Gaba FM Diouf A . Profile of plasma galectin-3 concentrations, inflammatory cytokines levels and lymphocytes status in breast cancer under chemotherapy. Open J Immunol. (2022) 12:1–14. doi: 10.4236/oji.2022.121001

[36] Zheng P Xiao Y Wu Z Wang Q Lv Y Niu W . Tumor-secreted galectin-3 suppresses antitumor response by inducing IL-10+ B cells. J Immunother Cancer. (2025) 13:e011445. doi: 10.1136/jitc-2024-011445, PMID: 40449954 PMC12142118

[37] Scafetta G D’Alessandria C Bartolazzi A . Galectin-3 and cancer immunotherapy: a glycobiological rationale to overcome tumor immune escape. J Exp Clin Cancer Res. (2024) 43:41. doi: 10.1186/s13046-024-02968-2, PMID: 38317202 PMC10845537

[38] Guo J-P Li X-G . Galectin-7 promotes the invasiveness of human oral squamous cell carcinoma cells via activation of ERK and JNK signaling. Oncol Lett. (2017) 13:1919–24. doi: 10.3892/ol.2017.5649, PMID: 28454344 PMC5403196

[39] Demers M Rose AAN Grosset A-A Biron-Pain K Gaboury L Siegel PM . Overexpression of galectin-7, a myoepithelial cell marker, enhances spontaneous metastasis of breast cancer cells. Am J Pathol. (2010) 176:3023–31. doi: 10.2353/ajpath.2010.090876, PMID: 20382700 PMC2877862

[40] Grosset A-A Poirier F Gaboury L St-Pierre Y . Galectin-7 expression potentiates HER-2-positive phenotype in breast cancer. PloS One. (2016) 11:e0166731. doi: 10.1371/journal.pone.0166731, PMID: 27902734 PMC5130216

[41] Trebo A Ditsch N Kuhn C Heidegger HH Zeder-Goess C Kolben T . High galectin-7 and low galectin-8 expression and the combination of both are negative prognosticators for breast cancer patients. Cancers (Basel). (2020) 12:953. doi: 10.3390/cancers12040953, PMID: 32290551 PMC7226378

[42] Wu G Deng W Chen H-Y Cho H-J Kim J . Galectin 7 leads to a relative reduction in CD4+ T cells, mediated by PD-1. Sci Rep. (2024) 14:6625. doi: 10.1038/s41598-024-57162-3, PMID: 38503797 PMC10951237

[43] Grosset A-A Labrie M Gagné D Vladoiu M-C Gaboury L Doucet N . Cytosolic galectin-7 impairs p53 functions and induces chemoresistance in breast cancer cells. BMC Cancer. (2014) 14:801. doi: 10.1186/1471-2407-14-801, PMID: 25367122 PMC4228062

[44] Nehmé R Fortier M Létourneau M Fuselier C Granger Joly de Boissel P Dumoulin A . Development of galectin-7-specific nanobodies: implications for immunotherapy and molecular imaging in cancer. J Med Chem. (2025) 68:8484–96. doi: 10.1021/acs.jmedchem.5c00071, PMID: 40208951 PMC12035796

[45] Laderach DJ Compagno D . Inhibition of galectins in cancer: Biological challenges for their clinical application. Front Immunol. (2023) 13:1104625. doi: 10.3389/fimmu.2022.1104625, PMID: 36703969 PMC9872792

